# Effects of Prior Thermal Exposure of a Soil–Compost System on Potentially Bioavailable Mercury and Its Accumulation in Rice Grown on Mining-Impacted Soils

**DOI:** 10.3390/toxics14080692

**Published:** 2026-08-05

**Authors:** Marisol Laza-Durante, Iván David Urango-Cárdenas, Germán Enamorado-Montes, Elvia Valeria Durante-Yánez, Roberth Paternina-Uribe, José Luis Marrugo-Negrete

**Affiliations:** 1 Water Research Group, Applied Chemistry and Environmental, Department of Chemistry, Faculty of Basic Sciences, Universidad de Córdoba, Carrera 6 No. 76-103, Montería 230002, Colombia; ivanurango@correo.unicordoba.edu.co (I.D.U.-C.); genamoradomontes@correo.unicordoba.edu.co (G.E.-M.); evdurante@correo.unicordoba.edu.co (E.V.D.-Y.); jmarrugo@correo.unicordoba.edu.co (J.L.M.-N.); 2 Faculty of Health Sciences, Universidad de Córdoba, Carrera 6 No. 76-103, Montería 230002, Colombia ; rpaternina@correo.unicordoba.edu.co

**Keywords:** potentially bioavailable mercury, soil–compost system, rice grain accumulation

## Abstract

Mercury (Hg) mobility in contaminated soils may be altered by solarization-induced thermal disturbance and organic amendments. This study evaluated whether a cover-induced prior solarization pretreatment of a mining-impacted soil–compost system modifies the operationally defined labile Hg fraction (F1 + F2, considered potentially bioavailable), rice productivity, grain Hg accumulation, and screening-level health risk. Mining soils from San Jorge River basin were amended with compost at a 1:9 ratio and solarized for one month before crop establishment, generating temperatures of 32, 40, 44, and 50 °C under different cover conditions. After pretreatment, covers were removed and rice was cultivated for four months under uniform flooded conditions. Total Hg in soils and grains was determined by EPA Method 7473, and the potentially bioavailable fraction by modified Bloom extraction. Prior thermal exposure increased this fraction, especially at 50 °C, whereas compost reduced total Hg and partially buffered this increase. Productivity depended on the compost × temperature interaction: compost improved yield at 40 and 44 °C, while no grain production occurred in substrates previously exposed to 50 °C. Compost-amended treatments showed lower grain Hg than unamended soils, although all values exceeded the 20 μg kg^−1^ reference limit. Hazard quotients remained below 1, with the highest value in unamended soil previously exposed to 44 °C.

## 1. Introduction

Thermal disturbances in agroecosystems can affect crop growth and yield and modify soil physicochemical and biogeochemical processes that regulate the mobility and availability of contaminants [1]. In agricultural systems, soil exposure to elevated temperatures, including those generated by management practices such as solarization, can disrupt the equilibrium between solid and soluble phases, modify oxidation-reduction processes, influence microbial activity, and transform the interaction between potentially toxic elements and soil organic matter. The combined effect of these changes is the potential increase in the bioavailable fraction of contaminants, thereby facilitating their incorporation into the food chain [2].

Among the potentially toxic elements of greatest concern is mercury (Hg), due to the diversity of its sources—both anthropogenic and natural—as well as its high toxicity, persistence, and ability to mobilize in the environment [3]. Furthermore, Hg has the potential for biomagnification in aquatic and terrestrial ecosystems, posing a significant threat to human health, ecological balance, and the sustainability of productive systems [4,5,6,7]. Hg bioavailability and mobility in soil are influenced by various factors, including temperature, precipitation, microbial transformation, complex formation, sorption, slow diffusion, and oxidation processes [8]. Of particular significance is the role of temperature, as it has been demonstrated to modify sorption-desorption equilibria, the chemical transformation of Hg, and the stability of organometallic complexes [9,10]. This, in turn, can result in alterations to its potentially bioavailable fraction. Consequently, exposure of soil to elevated thermal conditions may induce persistent changes in Hg dynamics, with subsequent effects on plant availability [11].

In order to reduce the mobility and bioavailability of potentially toxic elements in contaminated soils, various remediation strategies have been employed, including the incorporation of organic amendments such as compost [12,13]. This material enhances the physical and chemical properties of the soil, increases the amount of organic matter, promotes water retention, raises the pH in acidic soils, and provides functional groups that can immobilize metals through adsorption, complexation, or stabilization within the organic matrix [14,15]. Similarly, compost can function as a biologically active substrate, facilitating microbial transformations and retention mechanisms that reduce the availability of contaminants in the soil solution [16,17,18].

However, although compost has shown potential to reduce contaminant availability [19], it remains necessary to assess whether prior thermal exposure of the soil–amendment system, before crop establishment, can modify the efficacy of compost as a strategy for Hg immobilization. It has been demonstrated that thermal exposure has the capacity to modify the native organic matter present within the soil, as well as that which is introduced by the amendment. This phenomenon can also induce alterations in the stability of Hg-ligand complexes and other retention mechanisms, resulting in residual changes within the potentially bioavailable fraction of the metal [20,21]. Such modifications have the potential to persist after thermal treatment, thereby exerting an influence on Hg uptake by cultivated plants. This uncertainty is particularly relevant in rice-growing systems, as the flooding conditions characteristic of the crop alter Hg dynamics by changing the redox potential, the mobilization of dissolved organic matter, and the microbial transformation of the metal. Consequently, its availability in the rhizosphere and its uptake by the plant may be subject to alteration [22,23]. In this context, rice is not only a strategic crop for food security but also a potential route of human exposure to Hg through the accumulation of the metal in edible tissues [3,23,24,25]. Consequently, the establishment of the crop on soil–amendment systems previously exposed to elevated thermal conditions enables an assessment of whether the residual changes induced in Hg dynamics are reflected in increased transfer of the metal to the grain and, consequently, in a potential health risk. In this regard, prior thermal exposure via solarization was used as an experimental approach to generate contrasting thermal conditions in the soil–amendment system, without attempting to fully replicate the thermal regime experienced by a rice paddy throughout the entire growing cycle.

In Colombia, artisanal gold mining has been identified as a significant source of Hg release into the environment [26]. This phenomenon carries relevant implications for human health, particularly within vulnerable mining communities [27]. Within the San Jorge subregion of the Córdoba department, this issue is of particular concern due to the coexistence of areas affected by mining and rice-producing zones [28]; various studies have documented the presence of potentially toxic elements in this region [29,30,31,32,33]. Rice is a staple food of great importance in the local diet. According to the National Mechanized Rice Survey (ENAM), the weekly per capita consumption of rice in urban centers and rural areas of the department of Córdoba is approximately 1.37 kg, a figure that far exceeds the national average of 0.77 kg [34]. Consequently, any factor that increases the bioavailability of Hg in the soil and its uptake by crops could result in an additional food safety and public health problem in this region.

Despite the significance of this issue, there is limited information regarding the effect of prior solarization-induced thermal exposure of the soil–amendment system on the potentially bioavailable fraction of Hg in mining soils amended with compost, as well as on its subsequent accumulation in rice grains [2,21]. This knowledge gap limits our understanding of the stability of compost-based remediation strategies under experimental conditions of prior thermal disturbance and makes it difficult to anticipate their implications for food security in tropical regions affected by mining. In this context, the objective of this study was to evaluate the effect of cover-induced prior thermal exposure on the bioavailability of Hg in compost-amended mining soils, as well as to determine its subsequent accumulation in rice grains and estimate the associated screening-level health risk.

## 2. Materials and Methods

### 2.1. Study Area and Soil Sampling

The study area was situated within the San Jorge River basin, in the department of Córdoba, Colombia, within the area of influence of the Ayapel Wetland Complex. This area constitutes a component of the Integrated Management District (IMD) of the Ayapel Wetland Complex and was designated a Ramsar site on 23 June 2020. The selection of the area was based on the historical presence of artisanal gold mining and the influence of contaminated water inflows associated with this activity. As documented in previous reports, extractive activities have been developed in the area, with subsequent effects on soil and water components [35,36,37]. The region’s warm tropical climate is characterized by an average annual temperature of 28.1 °C, relative humidity levels ranging from 70 to 90%, and a bimodal precipitation pattern with an annual average of approximately 2500 mm. From a geological perspective, the predominant geological features in the region are sedimentary formations and alluvial soils [35]. 

**Figure 1:**
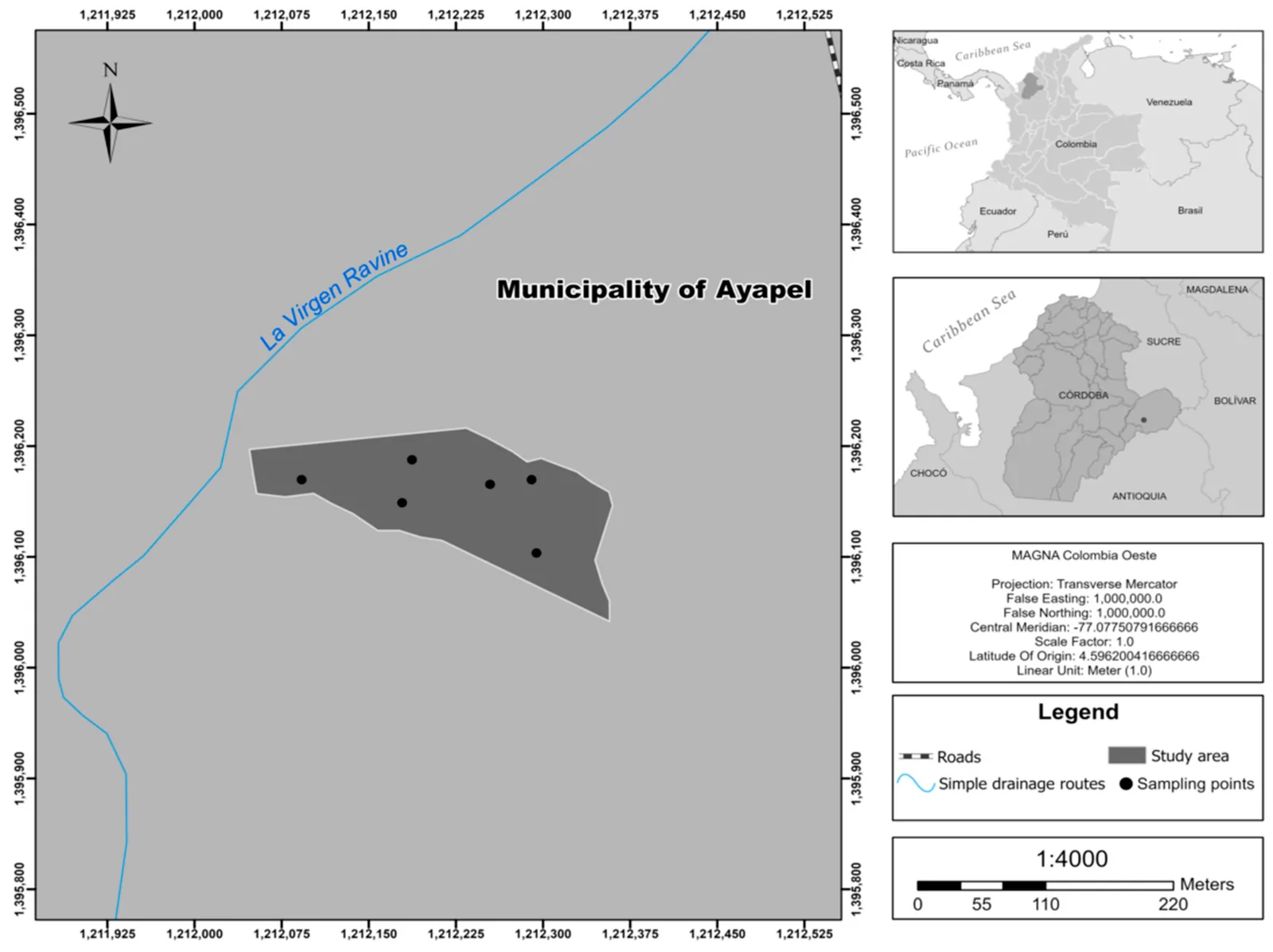
Study area located in Ayapel—Córdoba.

Six spot soil samples were collected from an artisanal gold mining-impacted area covering approximately 2.4 ha, corresponding to the shaded area shown in Figure 1. Sampling was conducted at a depth of 25 cm, following the removal of the surface layer, until approximately 500 kg of material were obtained for testing. The samples were then consolidated into a composite sample, which was subsequently transferred to a Screenhouse situated at the University of Córdoba, at 8°44′ N and 75°53′ W, at an elevation of 14 m above sea level, for the experimental phase.

### 2.2. Soil and Compost Preparation

The composite soil sample was subjected to a series of processing steps prior to the incorporation of compost and the experimental setup. These steps included air-drying, homogenization, and sieving to 4 mm [38]. Compost, comprising plant biomass, cattle manure, and organic food waste, was utilized as an organic amendment. The plant biomass was derived from phytoremediation processes of soils contaminated with heavy metals, a subject previously characterized by Caraballo-Laza et al. [14]. The experimental setup involved the incorporation of compost into soil at a volumetric ratio of 1:9 (compost:soil, *v*/*v*), as reported by Hu et al. [39]. Each soil–compost (SC) experimental unit contained 9 L of soil mixed with 1 L of compost, whereas each unamended soil (S) experimental unit contained 10 L of soil, resulting in the same final substrate volume (10 L) for both treatments. After mixing, the substrates were manually homogenized before the solarization pretreatment.

A completely randomized design with a 2 × 4 factorial arrangement was used. The first factor was compost application, with two levels: soil without compost (S) and soil with compost (SC). The second factor corresponded to cover-induced prior thermal exposure generated during the solarization pretreatment, with four mean diurnal thermal conditions: 32 °C (no cover), 40 °C (black cover), 44 °C (yellow cover), and 50 °C (transparent cover). Each treatment combination was established in triplicate, yielding a total of 24 experimental units. These thermal conditions were applied before crop establishment and should not be interpreted as rice-growing temperatures.

**Figure 2:**
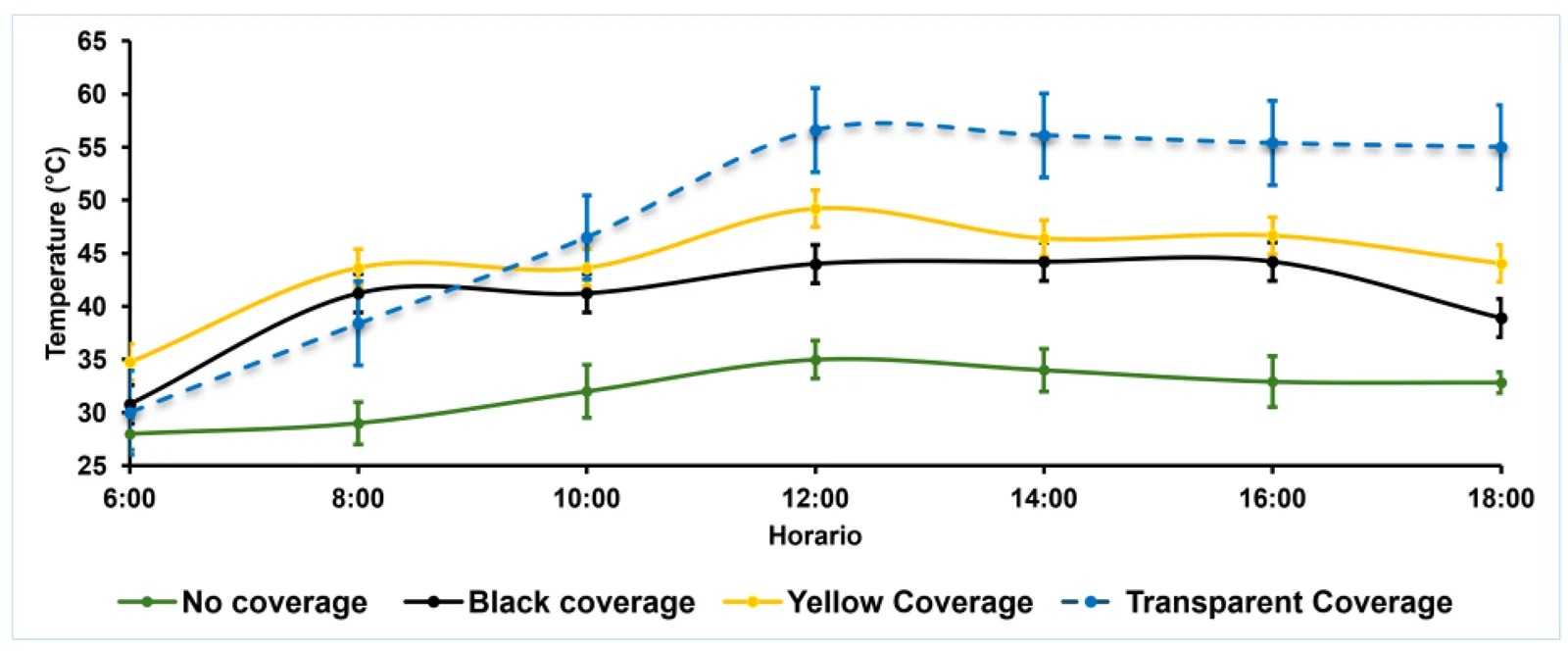
Mean soil temperature recorded at two-hour intervals during the solarization period.

The different thermal conditions were generated through solarization for a period of one month [40], prior to crop establishment, using plastic covers of different colors (black, yellow, and transparent), in addition to a treatment without cover as an environmental reference [41]. During the process of solarization, the soil or soil–amendment system was meticulously arranged in 15-cm-thick layers and maintained close to field capacity. The substrate was inspected and manually homogenized every two days to reduce moisture heterogeneity and was rewetted when necessary to prevent localized drying. This procedure was applied to promote more uniform heat transfer within the substrate during the pretreatment period. Soil temperature was monitored every two hours between 6:00 a.m. and 6:00 p.m. using a digital soil thermometer with a contact probe positioned within the upper 10 cm of the substrate. This configuration allowed continuous assessment of the thermal regime experienced throughout the 0–10 cm soil layer during the solarization treatment. The thermal gradient induced by the covers was determined by calculating daily averages for the experimental period and rounding the values to the nearest whole number. Consequently, four mean diurnal thermal conditions were established: 50 °C for the transparent cover, 44 °C for the yellow cover, 40 °C for the black cover, and 32 °C for the uncovered treatment (see Figure 2).

For the solarization pretreatment, one cover was used for each cover-induced thermal condition. After the pretreatment period, the covers were removed, and the conditioned substrates were used to establish the replicated rice cultivation experiment under uniform flooded conditions. This approach allowed the evaluation of the residual effects of prior solarization-induced thermal exposure of the soil–amendment system on Hg dynamics and subsequent accumulation in rice. Thus, the cover-induced thermal conditions were applied as a pre-crop substrate conditioning step and should not be interpreted as rice-growing temperatures or as independent effects of the plastic covers themselves. Since the thermal gradient was generated using plastic covers of different colors, the treatments may also have involved differences in microenvironmental conditions, including light transmission, evaporation, condensation, gas exchange, and microbial activity. Accordingly, the treatments were interpreted as cover-induced prior thermal exposure conditions rather than as isolated temperature treatments.

### 2.3. Establishment and Management of the Rice Crop

The experiment was conducted in 12-L containers using the commercial variety Fedearroz 2020 (FA2020), which was supplied by the National Federation of Rice Growers of Colombia [42]. The seeds were pre-selected by subjecting them to flotation in distilled water for a period of two minutes, after which the seeds that remained on the surface were discarded. Approximately 10 viable seeds were sown in each experimental unit [24]. The crop was maintained under flooding conditions, with a water depth of approximately 3–5 cm, which was periodically adjusted to compensate for losses due to evaporation. Fertilization was applied uniformly across all treatments at field-equivalent rates of 120 kg N ha^−1^, 60 kg P_2_O_5_ ha^−1^, and 60 kg K_2_O ha^−1^, corresponding to approximately 0.59 g N, 0.29 g P_2_O_5_, and 0.29 g K_2_O per experimental unit. Phosphorus and potassium were applied at crop establishment, whereas nitrogen was split into three applications: 0.24 g N at the onset of tillering, 0.18 g N during active tillering, and 0.18 g N at the beginning of reproductive differentiation. Weed control was performed manually, and crop health was monitored periodically, with corrective plant protection measures applied only when necessary. At the end of the growing cycle, flooding was stopped to promote grain maturation and drying before harvest. The trial lasted four months. At the end of the cycle, the number of filled grains, the number of empty grains, and the total grain weight per experimental unit were recorded.

### 2.4. Determination of Soil Physicochemical Properties

To characterize the experimental substrates, representative samples were collected, air-dried, and stored in polyethylene bags until analysis. The pH was determined by potentiometry (NTC 5264: 17-10-2018 [43], the total organic carbon by titrimetry (NTC 5403:19-05-2021) [44], the available sulfur by turbidimetry (NTC 5402: 22-02-2006 [45] and the available phosphorus by colorimetry (NTC 5350:09-09-2020) [46]. Exchangeable bases (Ca, Mg, K, and Na) were determined by atomic absorption spectrophotometry and emission spectrometry, using 1 M ammonium acetate at pH 7.0 (PLSA-016, V.07 of 27-05-2022) [47]. The effective cation exchange capacity (ECC) and the sum of cations were calculated from the concentrations of the exchangeable bases. Exchangeable acidity was determined using 1.0 M KCl (NTC 5263: 21-06-2017) [48], while the textural class was established using the Bouyoucos or hydrometer method [49].

### 2.5. Determination of Total Hg and the Potentially Bioavailable Fraction in Soils

To determine the total mercury (HgT) content, approximately 500 g of soil samples were collected at the beginning and end of the experiment. The samples were dried at room temperature and homogenized by grinding and sieving to 2 mm. The concentration of total mercury (HgT) in soils was determined using a Milestone DMA-80 direct Hg analyzer (Milestone S.r.l., Sorisole, Italy), in accordance with EPA Method 7473 [50].

The operationally defined labile Hg fraction in soils was estimated using a modification of the sequential extraction method described by Bloom et al. [51]. According to the five fractions outlined by this method, the sum of the water-soluble fraction (F1 or F-w), obtained with deionized water, and the weak acid-soluble fraction (F2 or F-h), extracted with 0.01 M HCl + 0.1 M CH_3_COOH, was considered an operationally defined labile Hg fraction, here interpreted as potentially bioavailable. This fraction should not be interpreted as a direct measurement of plant-available Hg. For this extraction, 1 g of soil was used and the final extract volume was adjusted to 25 mL. Hg in the extracts was quantified using an RA-915M Hg analyzer coupled (Lumex Instruments, St. Petersburg, Russia), with the RP-92 module and a multipath analytical cell. Quality control included procedural blanks, reagent blanks, and replicate analyses. Under these analytical conditions, the instrumental detection limit was 12 ng L^−1^, corresponding to a method detection limit of 0.300 µg kg^−1^ on a soil dry-weight basis. The corresponding limit of quantification was 1.00 µg kg^−1^.

Analytical quality control for Hg determination in soils was performed in triplicate using the certified reference material SQC001—Metals in soils (Sigma Aldrich, St. Louis, MO, USA) (Hg = 4.59 ± 0.0778 mg/kg). The method demonstrated good precision, with CV values ranging from 4.8 to 6.3%, and satisfactory accuracy, with recoveries of 97–105% for spiked samples and 96–103% for the certified reference material. The method detection limit (LOD) for Hg in soils samples was 0.73 µg kg^−1^, calculated as three times the standard deviation of procedural blanks (*n* = 10), while the limit of quantification (LOQ) was 2.43 µg kg^−1^, calculated as ten times the standard deviation of the blanks.

At the end of the growing cycle, the rice grains were harvested and prepared for analysis. The concentration of total Hg in the grains was determined using a Milestone DMA-80 direct Hg analyzer, following EPA Method 7473 [50]. The analytical quality of Hg determination in plant samples was assessed using the certified reference material Trace and Minor Elements in Lichen (IAEA-336; International Atomic Energy Agency, (IAEA), Vienna, Austria). (*n* = 3). Precision was deemed satisfactory, with coefficients of variation ranging from 1.2 to 10.0%. Accuracy was evaluated through recovery assays, with values ranging from 98.0 to 99.5% for the certified reference material (CV < 2%). The detection limit (LOD) for Hg in plant samples was 0.71 µg kg^−1^, calculated as three times the standard deviation of the blanks (*n* = 10), while the limit of quantification (LOQ) was 2.40 µg kg^−1^, calculated as ten times the standard deviation of the blanks.

### 2.6. Assessment of Hg Exposure Using Estimated Weekly Intake (EWI) of Rice

The assessment of the health risk associated with Hg exposure through rice consumption is quantified by estimating the Estimated Weekly Intake (*EWI*) of Hg [32]. It is expressed in micrograms of Hg per kilogram of body weight per week (μg kg^−1^ body weight week^−1^) and is calculated using the following equation (Equation (1)):

Equation (1): Estimated Weekly Intake

(1) $$EWI=C\times \frac{IR}{BW}$$

where *C* is the average concentration of the element in the grain for each treatment (μg g^−1^); *IR* is the weekly rice consumption rate (kg week^−1^), which for the department of Córdoba is 1.374 kg week^−1^ according to the National Survey of Mechanized Rice (ENAM) [34]; and *BW* is body weight (kg), where 70 kg was used as a reference.

### 2.7. Non-Carcinogenic Risk Assessment for Hg Exposure: Hazard Quotient (HQ)

The *HQ* is a tool used to assess the potential risk to human health associated with exposure to non-carcinogenic contaminants, such as Hg. This indicator makes it possible to determine whether exposure to a chemical substance may pose a significant health risk. It is calculated using the following equation (Equation (2)):

Equation (2): hazard quotient

(2) $$HQ=\frac{EWI}{PTWI}$$

where the provisional tolerable weekly intake (*PTWI*) for Hg is 4 μg Hg kg^−1^ body weight week^−1^, according to JECFA [52]. In this context, an *HQ* ratio < 1 indicates no risk of non-carcinogenic effects, while an *HQ* > 1 indicates the possibility of non-carcinogenic effects occurring.

The *PTWI* applied in this study corresponds to the historical value established by JECFA for total mercury. However, this guideline has subsequently been withdrawn because methylmercury is now recognized as the toxicologically most relevant Hg species in food exposure. Since only total Hg was quantified in rice grains, the risk assessment should be interpreted as a screening-level evaluation rather than a definitive assessment of human health risk. The actual risk may differ depending on the proportion of total Hg present as methylmercury.

### 2.8. Statistical Analysis

The data were organized in Microsoft Excel from the Office 365 suite and analyzed using R software version 4.3.0 and RStudio version 2023.03.1+446. Data normality was assessed using the Shapiro–Wilk test, and values with *p* > 0.05 were considered to meet the normality assumption. Homogeneity of variances was evaluated using Levene’s test. For the factorial design, a two-way ANOVA was performed to evaluate the effects of compost application, prior solarization-induced thermal exposure, and their interaction. When the compost × thermal exposure interaction was significant, treatment combinations were compared and interpreted rather than only the main effects. When significant differences were detected, multiple comparisons were conducted using Tukey’s HSD test, with a significance level of α = 0.05. For variables associated with grain yield and Hg concentration in grain, treatments previously exposed to 50 °C were excluded from the inferential analysis due to the absence of harvestable grain; therefore, these analyses were performed as a 2 × 3 factorial arrangement.

## 3. Results and Discussion

### 3.1. Characterization of the Compost

In accordance with NTC 5167:2022, the compost used as an amendment exhibited favorable physical and chemical characteristics for incorporation into the experimental system (Table 1). The material had a moisture content of 24.8%, a pH of 7.25, a cation exchange capacity of 41 meq 100 g^−1^, a bulk density of 0.54 g cm^−3^, total oxidizable organic carbon of 15.9%, and a C/N ratio of 17.7, values consistent with a stabilized organic material with the potential to improve substrate properties. Likewise, moisture retention was 132.6% and ash content was 16.6%, both within the criteria established by the standard.

In the mineral analysis, the compost had a total potassium content of 1.55%, while the Hg content was 0.17 mg kg^−1^, a value well below the maximum allowed by NTC 5167:2022. However, total nitrogen (0.79%) and total phosphorus (0.58%) were below the minimum reference values, suggesting limited capacity as a source of these nutrients. Overall, the results indicate that the compost used was suitable as an organic amendment to modify the properties of the soil–amendment system, with a low risk of introducing additional Hg into the experiment.

Based on these characteristics, the incorporation of compost allowed for the formation of a soil–amendment system with chemical properties distinct from those of the untreated soil. Consequently, the initial physicochemical properties of the experimental substrates were evaluated to identify changes associated with the addition of the amendment prior to thermal exposure and crop establishment.

### 3.2. Initial Physicochemical Properties of the Experimental Substrates

The soil texture was classified as sandy loam and remained unchanged in the treatments without compost (S) and with compost (SC), indicating that the addition of the amendment did not alter the textural class [53]. Such textures typically have low macro- and micronutrient contents, so these soils are considered depleted [54]. In general, these substrates have limitations in terms of fertility, organic matter content, and water-holding capacity [55]. Furthermore, their high sand content suggests high activity of potentially toxic elements in the soil solution [56].

The control soil (S) exhibited acidic conditions (pH) [57], and an exchangeable acidity (Al + H) of 2.42 cmolc kg^−1^. Levels above approximately 1 cmolc kg^−1^ can be phytotoxic to plants [58]. In contrast, the SC treatment showed a near-neutral pH (7.30), higher organic matter content (1.87%), and higher concentrations of sulfur, phosphorus, calcium, magnesium, potassium, and sodium, all of which are essential plant nutrients. Furthermore, no exchangeable acidity was detected in the SC treatment.

**Figure 3:**
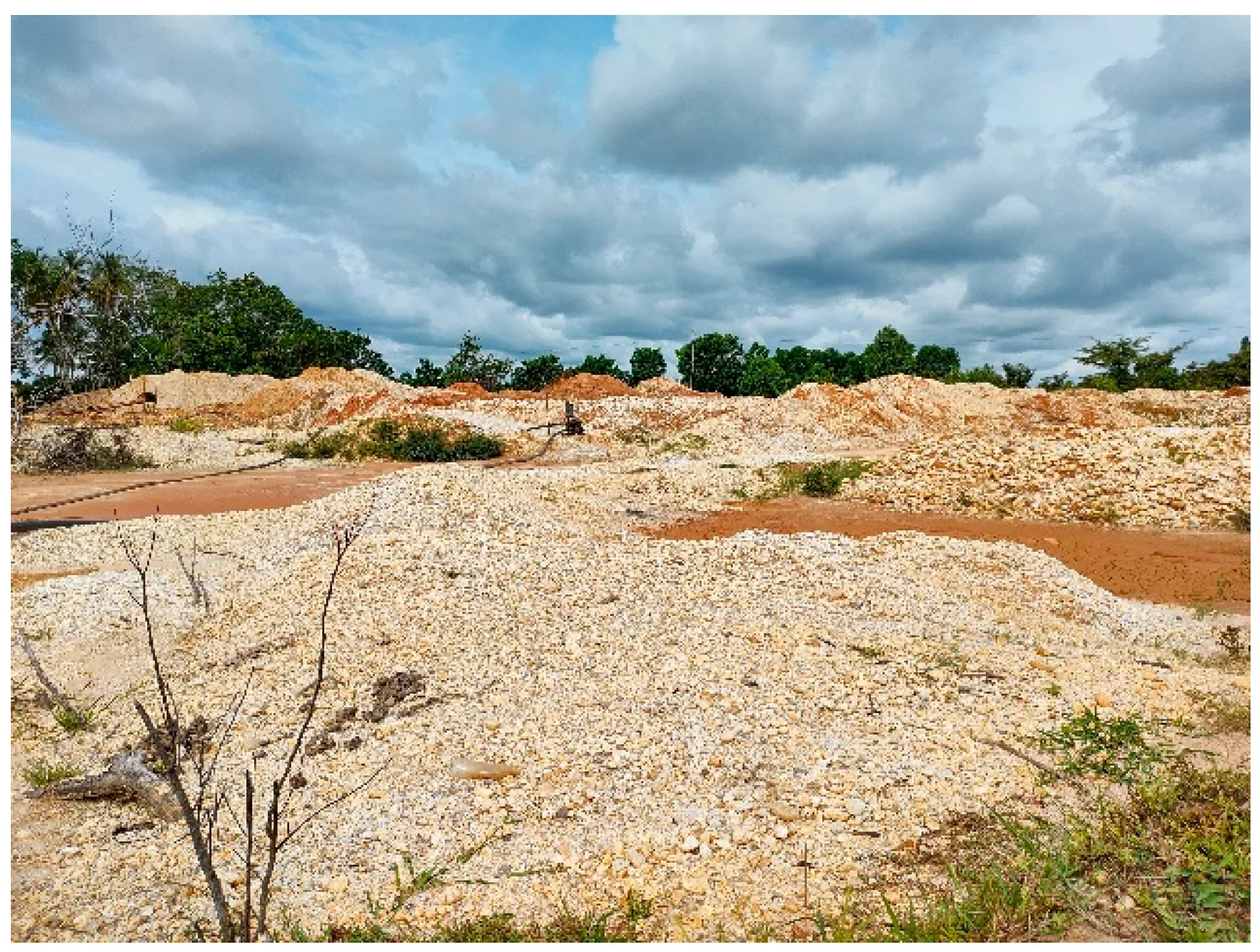
Soil in the study area, lacking organic horizons and with a high presence of rocks.

The soil conditions reported for the S treatment (control) are typical of soils affected by mining activities, where a loss of organic and surface horizons is observed, with a high presence of sand, stones, and rocks (Figure 3). These findings are consistent with those reported in studies conducted in areas previously degraded by mining activity [59].

The application of compost resulted in an increase in organic matter (OM) and nutrients such as phosphorus (P), calcium (Ca), magnesium (Mg), potassium (K), and sulfur (S) (Table 2). This process improved soil fertility and contributed to a significant increase in effective cation exchange capacity (ECEC), which rose from 3.1 to 18.2 cmol(+) kg^−1^. This relatively high ECEC indicates an improved capacity of the amended substrate to retain exchangeable cations. Although mining soils are frequently characterized by poor fertility, the observed increase in ECEC suggests that compost enhanced cation adsorption capacity, which may influence both nutrient availability and Hg retention [60]. This response is consistent with the supply of macro- and micronutrients and organic functional groups provided by compost [61]. Organic amendments can improve the physical and chemical condition of degraded soils because they provide stable carbon, reactive surfaces, and negative surface charges [53,62]. Studies such as those conducted by Munera-Echeverry et al. [63] in tropical soils have demonstrated that organic amendments have a positive effect on soil fertility, including increased K content, water-holding capacity, pH, and ECEC. The presence of macro- and micronutrients in mature compost is attributed to the source materials used in the composting process [64].

### 3.3. Cover-Induced Thermal Conditions Reached During Solarization of the Soil–Amendment System

Solarization of the soil–amendment system allowed the establishment of a cover-induced thermal gradient among the evaluated treatments (Figure 2). Based on the daily averages recorded during the month of exposure, four mean diurnal thermal conditions were identified: transparent cover (50 °C) > yellow cover (44 °C) > black cover (40 °C) > no cover (32 °C). These results confirm that the use of plastic covers of different colors was effective in generating thermal contrasts in the experimental system prior to crop establishment.

The highest thermal condition was recorded under the transparent cover, with an increase of 18 °C compared with the no-cover treatment, whereas the yellow and black covers generated intermediate increases of 12 and 8 °C, respectively. These results indicate that the thermal response of the system was associated with the type of cover used, allowing the establishment of differentiated prior solarization conditions to evaluate their residual effects on Hg dynamics. However, because plastic covers may also modify light transmission, evaporation, condensation, gas exchange, and microbial activity, the observed responses should be interpreted as effects of cover-induced solarization pretreatment rather than temperature alone. The color of the plastic cover influences the absorption and transmission of radiant energy, thereby modifying the microclimatic conditions surrounding the soil and the resulting substrate temperature [40].

It should be noted that these temperatures apply exclusively to the thermal exposure phase prior to crop establishment, under conditions of exposed soil. Therefore, they do not directly represent the soil temperature regime during rice growth, since plant cover and subsequent management under flooded conditions tend to dampen the system’s thermal response. Consequently, the treatments should be interpreted as cover-induced prior thermal exposure conditions aimed at evaluating residual effects on the bioavailability of Hg and its subsequent transfer to the crop.

### 3.4. Initial Total and Potentially Bioavailable Hg Concentrations in Mining Soils Amended with Compost

The substrates evaluated, originating from soils affected by small-scale artisanal gold mining, exhibited appreciable Hg contents. The accumulation and behavior of this element in the soil are influenced by physical and chemical properties such as acidity, organic matter composition, and texture [65]. According to Table 3, initial total Hg concentrations were significantly lower in the compost treatment (SC) than in the treatment without amendment (S), according to Welch’s independent-samples *t*-test (t = 5.23, df = 3.10, *p* = 0.047). This reduction may be associated with the dilution effect generated by the incorporation of compost, as well as changes in the chemistry of the soil–amendment system related to increased pH and organic matter, factors that influence Hg retention and distribution in the substrate [66,67]. Furthermore, although these values were lower than the maximum levels reported for gold mining areas with localized Hg accumulation, they exceed national and international reference values for uncontaminated soils. In this same area of Ayapel, De la Rosa-Mendoza et al. [68] reported total Hg concentrations ranging from 40.8 to 52,044.4 µg kg^−1^, with a mean value of 1680.6 µg kg^−1^, highlighting the high variability of Hg in soils affected by mining. Likewise, the values recorded in the present study exceed the background value of 51 ± 3 µg kg^−1^ calculated for non-agricultural soils in other areas of Colombia [69] and the reference value of 28 ± 7 µg kg^−1^ proposed by Marrugo-Negrete et al. [70]. for uncontaminated Colombian soils. Furthermore, they fell within the reference range reported by UNEP for contaminated soils (70–300 µg kg^−1^) [71], confirming the presence of Hg contamination in the experimental material.

Unlike total Hg, the initially potentially bioavailable fraction was significantly higher in the SC treatment than in the S treatment (t = −7.65, df = 4.00, *p* = 0.0016). This result suggests that, although the incorporation of compost decreased the total concentration of the metal, it may also have initially favored the presence of more labile or operationally extractable Hg forms, possibly due to changes in organic matter composition, the release of soluble ligands, or the redistribution of Hg among geochemical fractions within the soil–amendment system [66]. In this regard, the initial results indicate that compost addition not only modified the total amount of Hg present in the substrate but also its partitioning among geochemical fractions with different degrees of lability. This initial redistribution provides an important basis for interpreting the subsequent effects of cover-induced thermal exposure on Hg dynamics and the observed patterns of Hg accumulation in rice plants.

### 3.5. Total Hg and the Potentially Bioavailable Fraction in Substrates Following Thermal Exposure of the Soil–Amendment System

**Figure 4:**
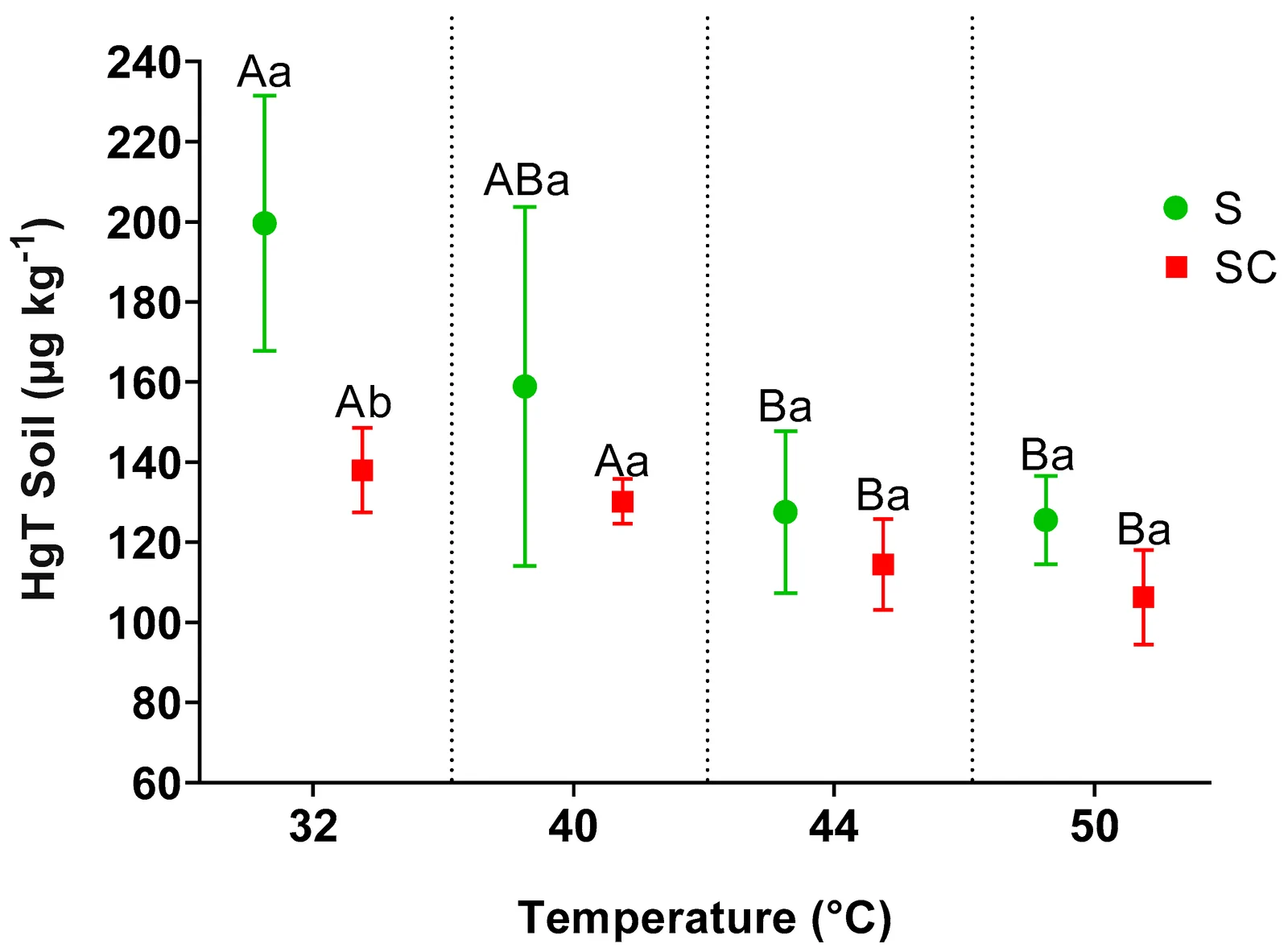
Total Hg concentration in substrates after prior thermal exposure of the soil–amendment system. Lowercase letters indicate significant differences between S and SC within the same thermal condition. Uppercase letters indicate significant differences among thermal conditions within the same substrate treatment. Differences were determined using Tukey’s HSD test (*p* < 0.05). S: soil without compost; SC: soil with compost.

Analysis of total Hg (HgT) concentrations in the substrates following thermal exposure of the soil–amendment system revealed significant differences for compost application (F_1,16_ = 11.41, *p* = 0.0038), prior thermal exposure, and the compost × temperature interaction (F_3,16_ = 8.49, *p* = 0.0026), according to two-way ANOVA and Tukey’s test (Figure 4). The significance of this interaction indicates that the effect of compost on HgT concentration depended on the solarization condition to which the soil–amendment system was previously exposed. The highest HgT concentration was recorded in the treatment without compost (S) under the 32 °C condition, with a value of 199.65 ± 31.85 µg kg^−1^, while the lowest concentration was observed in the compost treatment (SC) exposed to 50 °C, at 108.07 ± 8.52 µg kg^−1^. In general, the compost treatments exhibited lower HgT concentrations than the soil without amendment, suggesting that the incorporation of compost altered the metal dynamics in the experimental system, although the magnitude of this effect varied depending on the intensity of the prior exposure.

When comparing the initial HgT concentrations with those recorded after solarization, a decrease of between 13 and 31% was observed in the evaluated treatments. This behavior could be associated with changes in Hg retention within the soil–amendment system and, possibly, with losses due to volatilization favored by high thermal conditions or with redistribution within the substrate [68]. The results indicate that prior solarization generated persistent changes in the behavior of total Hg in the substrates, even after the covers were removed and before crop establishment. The total Hg concentrations obtained were found to be lower than those reported by Cherian et al. [72] at a Ramsar site in Kerala, India.

**Figure 5:**
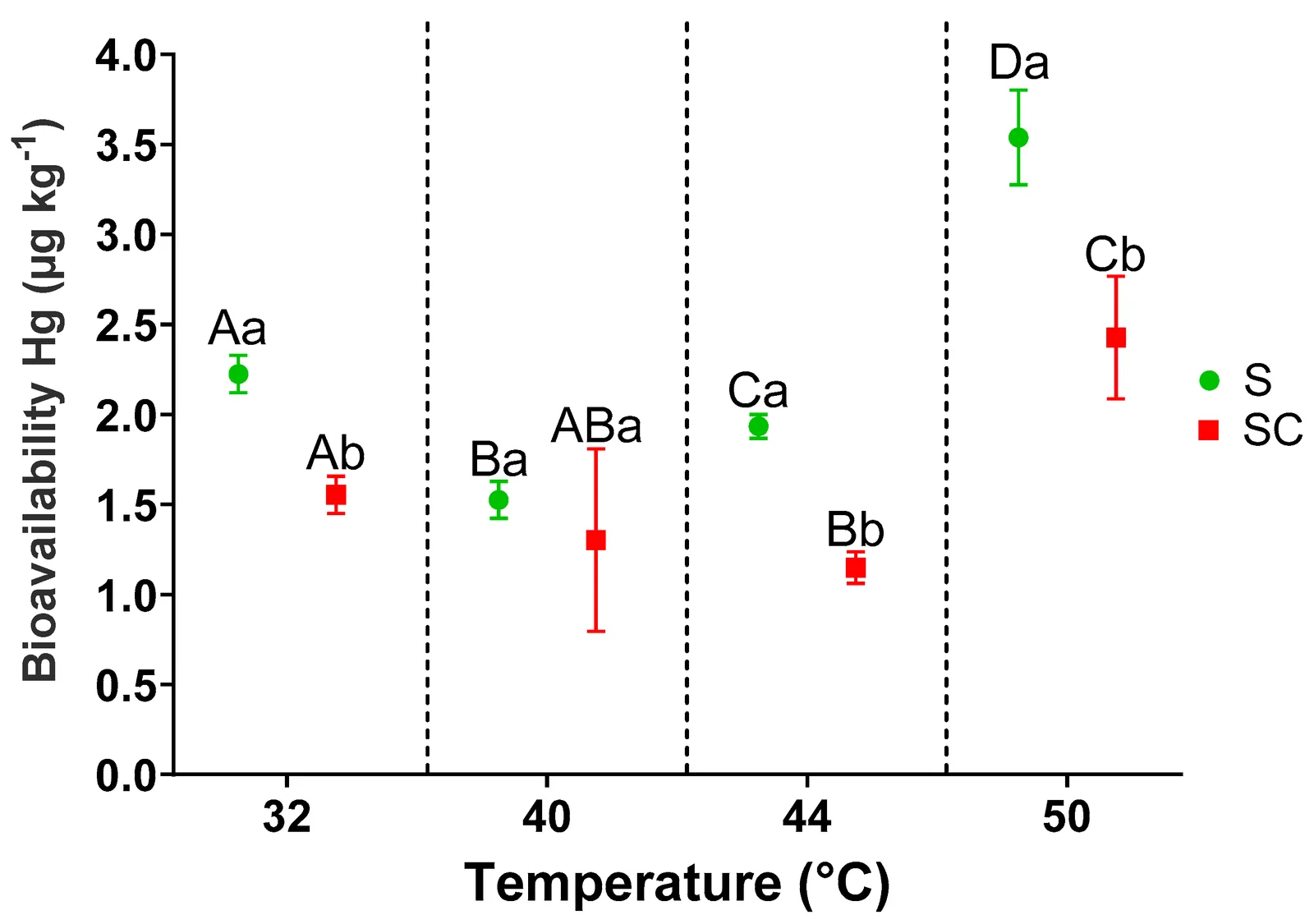
Potentially bioavailable Hg fraction in substrates after prior thermal exposure of the soil–amendment system. Lowercase letters indicate significant differences between S and SC within the same thermal condition. Uppercase letters indicate significant differences among thermal conditions within the same substrate treatment. Differences were determined using Tukey’s HSD test (*p* < 0.05). S: soil without compost; SC: soil with compost.

Regarding the potentially bioavailable fraction of Hg, the results showed significant differences for compost application (F_1,16_ = 71.6; *p* < 0.0001), prior thermal exposure, and the compost × temperature interaction (F_3,16_ = 10.69; *p* = 0.045) (Figure 5). This interaction indicates that the effect of the amendment on the potentially bioavailable fraction was not constant but depended on the cover-induced thermal condition. In general, the incorporation of compost reduced this fraction compared to unamended soil after solarization,, suggesting an immobilizing effect associated with the organic matter provided by the amendment, particularly under conditions of lower thermal exposure [66]. However, the highest values of the potentially bioavailable fraction were recorded at 50 °C, indicating that more intense thermal disturbance favored the release of Hg into more labile or operationally available forms [73]. Despite this increase, the SC treatment exhibited lower values than S under most of the evaluated conditions, possibly due to complexation and adsorption processes of Hg onto compost components [67]. Taken together, these results suggest that compost exerted a buffering effect against the increase in the potentially bioavailable fraction induced by thermal exposure, although this effect was reduced under the highest thermal condition.

When the initial operationally defined labile Hg fraction (F1 + F2) was compared with that measured after solarization, the largest increases were observed at 50 °C, where concentrations reached approximately 3.54 µg kg^−1^ in S and 3.65 µg kg^−1^ in SC. These values represented increases of 254% and 192%, respectively, compared with the initial concentrations of 1.00 µg kg^−1^ in S and 1.25 µg kg^−1^ in SC. Despite these increases, the F1 + F2 fraction remained a small proportion of total Hg, generally below 2%. Therefore, the observed pattern indicates a redistribution of Hg toward operationally extractable forms after solarization, without implying a direct measurement of plant-available Hg. The lower increase observed in SC suggests that compost partially attenuated the redistribution of Hg toward more labile forms. This response may reflect a progressive shift from the initial mobilization of Hg by soluble organic ligands to partial immobilization through adsorption, complexation, chelation, and enhanced cation retention associated with the higher pH, organic matter content, and ECEC of the amended substrate [73,74,75,76,77,78].

### 3.6. Yield Response of Oryza sativa L. to Compost and Prior Solarization-Induced Thermal Exposure

**Figure 6:**
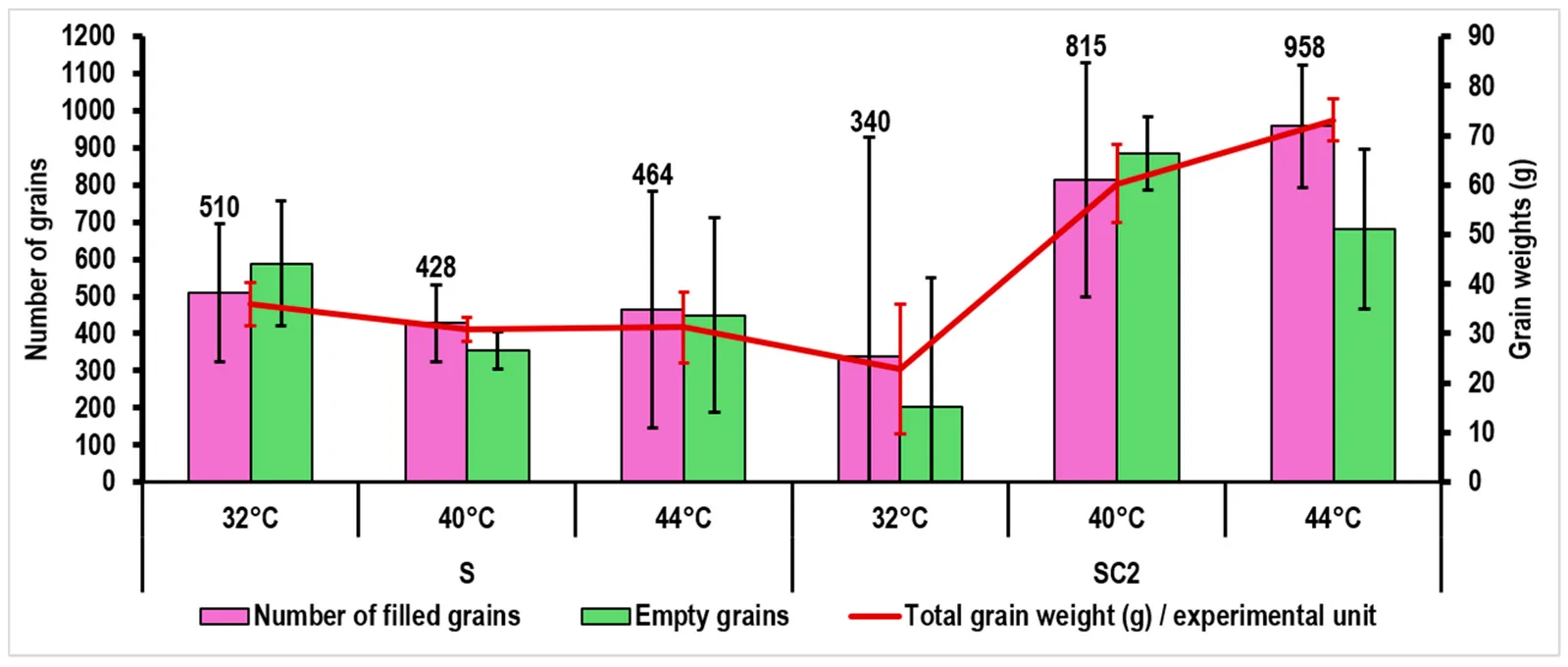
Number of filled grains, empty grains, and total grain weight per experimental unit obtained at the end of the trial. S: soil without compost (control); SC: soil + compost.

According to Figure 6, descriptive variations were observed among the substrate × solarization combinations regarding the number of filled grains, number of empty grains, and total grain weight; however, the statistical responses differed among these variables. The two-way ANOVA revealed no significant main effects of solarization or its interaction with compost on the number of filled grains (solarization: F_2,12_ = 1.36; *p* = 0.294; compost × solarization: F_2,12_ = 2.02; *p* = 0.175); conversely, a significant effect was exerted by the compost amendment (F_1,12_ = 8.68; *p* = 0.048). For the number of empty grains, neither compost amendment nor solarization treatment displayed significant main effects, whereas their interaction was significant F_2,12_ = 7.08; *p* = 0.009), indicating that the influence of compost depended on the soil-cover treatment and its associated thermal conditions. In contrast, total grain weight was significantly affected by both individual factors and their interaction: compost amendment (F_1,12_ = 30.77; *p* < 0.001), solarization (F_2,12_ = 14.72; *p* < 0.001), and compost × solarization (F_2,12_ = 22.67; *p* < 0.001).

In treatments previously solarized under the transparent plastic cover, which reached approximately 50 °C, no grain production was recorded; therefore, this condition was not included in the final yield comparison. The absence of grains in treatments previously exposed to 50 °C indicates that extreme thermal exposure may have generated negative residual effects on the soil–amendment system’s ability to support the crop’s reproductive phase. Although the plants were not directly subjected to 50 °C during their development, this condition may have altered substrate properties related to nutrient availability, microbial activity, and the stability of organic matter [79,80,81]. Furthermore, under high-temperature conditions, the labile organic matter in compost can decompose rapidly, leading to potential nitrogen losses through volatilization or transformations that reduce the crop’s nutrient use efficiency [82,83]. Additionally, when compost lacks adequate stability or is subjected to intense thermal exposure, intermediate organic compounds with phytotoxic potential may be generated, which could affect plant establishment and subsequent development [83,84].

In rice systems, this response may be even more complex, as subsequent crop flooding depends on a functional microbiota capable of regulating redox processes, nutrient transformation, and the dynamics of gases such as CH_4_; therefore, intense thermal disturbance of the substrate could alter the microbiological balance before rice establishment.

The literature on heat stress in cereals shows that high temperatures can affect morphophysiological and reproductive processes, including pollination, fertilization, grain number, and grain filling, and that the extent of the damage depends on the intensity and duration of exposure [85]. This result suggests that, under extreme thermal conditions, the ability of compost to improve yield performance may be limited.

Rice yield was influenced by the interaction between compost amendment and the prior solarization treatment generated by the different soil covers, particularly for total grain weight. Under the reference condition without solarizing cover, associated with a soil temperature of approximately 32 °C, no significant difference in total grain weight was observed between S and SC (*p* = 0.102). The mean value was numerically lower in SC than in S, indicating that compost incorporation alone did not produce a statistically detectable improvement under this condition. Likewise, no significant differences in grain weight were observed among the solarization conditions within the unamended substrate. In contrast, under the covers associated with soil temperatures of approximately 40 and 44 °C, the compost-amended substrate produced significantly greater grain weights than the corresponding unamended substrate. Although the number of empty grains also exhibited a significant substrate × solarization interaction, this variable should not be interpreted as an indicator of improved productive performance. However, the improvement in productive performance should be mainly supported by the higher number of filled grains and grain weight. The highest number of filled grains was recorded in SC at 44 °C, with an average of 958 grains, compared with 464 grains obtained in S under the same solarization condition.

This behavior suggests that the favorable effect of compost depended on the soil conditions generated by the different solarization covers and was most evident under the treatments associated with soil temperatures of approximately 40 and 44 °C. A possible explanation is that moderate thermal exposure may have favored processes of organic matter mineralization and nutrient release in the compost-treated system, improving substrate conditions for subsequent crop development [84]. In contrast, under the 32 °C condition, the effect of the amendment was less evident, while at 50 °C thermal exposure may have exceeded the system’s tolerance threshold, limiting grain formation. This pattern suggests a nonlinear response of the soil–amendment system, in which moderate prior thermal exposure may have enhanced nutrient mineralization in compost-amended substrates, whereas extreme exposure at 50 °C likely exceeded the functional capacity of the substrate to support reproductive development [86].

In general terms, it is known that low levels of macro- and micronutrients tend to reduce grain weight, negatively impacting plant yield [87]. Furthermore, the use of organic amendments leads to increased growth; however, this increase is linked to the physical and chemical properties of the soil in which the plants grow [88,89]. Taking the above into account, the effect of compost application can be considered positive, as the plants achieved better productive development, particularly under the initial thermal conditions of 40 and 44 °C. These plants were not only exposed to Hg but also grown in substrates with low fertility, low organic matter, acidity, and sandy texture, which are characteristic of soils affected by mining activities [90].

### 3.7. Hg Concentration in O. sativa Grains

**Figure 7:**
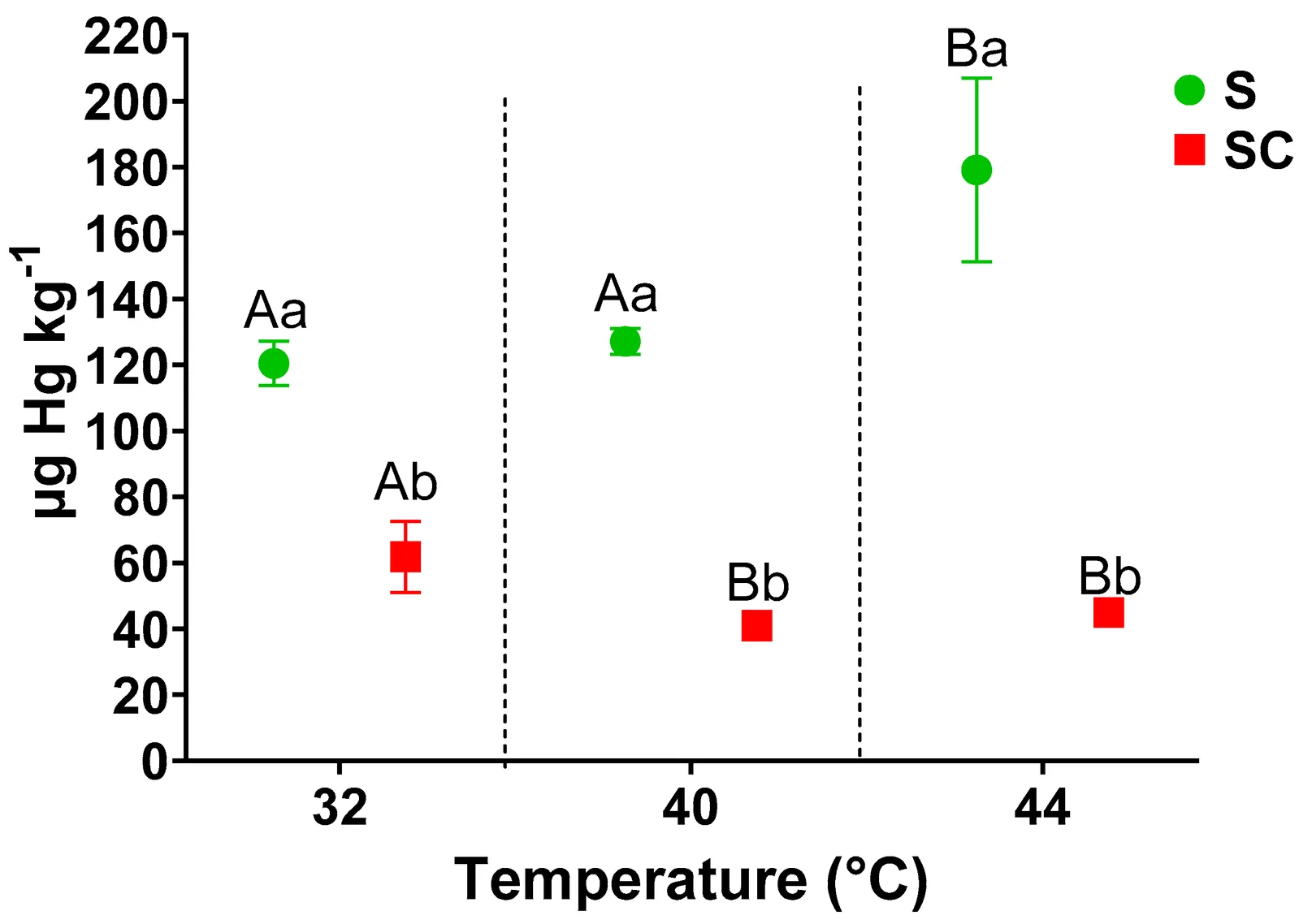
Total Hg concentration in *Oryza sativa* L. grains grown in substrates with and without compost after prior thermal exposure of the soil–amendment system. Lowercase letters indicate significant differences between S and SC within the same thermal condition. Uppercase letters indicate significant differences among thermal conditions within the same substrate treatment. Differences were determined using Tukey’s HSD test (*p* < 0.05). Treatments previously exposed to 50 °C were not included because no harvestable grains were produced. S: soil without compost; SC: soil with compost.

The two-way ANOVA revealed significant effects of substrate (F_1,12_ = 240.66, *p* < 0.0001), solarization treatment (F_2,12_ = 7.87, *p* = 0.0066), and their interaction (F_2,12_ = 13.54, *p* = 0.0008) on Hg concentration. The significance of this interaction indicates that the effect of compost on Hg accumulation in the grain depended on the prior solarization condition to which the soil–amendment system was subjected. Tukey’s test for comparing means confirmed differences among the evaluated treatments. In general, Hg concentrations in the grains were significantly lower in the compost treatments (SC) than in the soil without amendment (S), suggesting that the incorporation of compost helped reduce the accumulation of the metal in the edible tissue of the rice (Figure 7).

The highest Hg concentration in grains was recorded in the control treatment (S) previously exposed to 44 °C, with 179.1 μg kg^−1^, whereas the lowest concentration was observed in the compost-amended treatment (SC) previously exposed to 40 °C, with 40.13 μg kg^−1^ (Figure 7). The results obtained were higher than those reported by Enamorado-Montes et al. [24], who found grain concentrations of 8.64 μg Hg kg^−1^ in commercial rice varieties in the La Mojana region. However, previous studies in the region were generally conducted under agricultural soil conditions and did not evaluate the operationally defined labile Hg pool in degraded mining substrates [24]. In the present study, the soils were collected from areas strongly affected by artisanal gold mining and can be considered environmental liabilities of historical mining activity. Their low organic matter content, greater acidity, and sandy loam texture may reduce Hg retention capacity and favor contaminant redistribution and mobility under flooded cultivation conditions.

To further examine Hg accumulation in rice grains, the soil-to-grain Hg bioaccumulation factor (BAFgrain) was calculated as the ratio between total Hg concentration in rice grain and total Hg concentration in the corresponding substrate. In unamended soils (S), BAFgrain increased from 0.60 at 32 °C to 0.80 at 40 °C and reached 1.40 at 44 °C, indicating high apparent accumulation of Hg in edible tissue relative to total Hg in the substrate. In contrast, compost-amended treatments (SC) showed lower and more stable BAFgrain values, ranging from 0.31 to 0.45. Although the BAFgrain value of 1.40 may appear high for total Hg, previous studies in Hg-contaminated rice-growing areas have reported BAF values greater than 1 for methylmercury in rice grains, whereas BAF values for inorganic Hg are generally lower than 0.5 [91]. This indicates that high Hg accumulation ratios in rice grain can occur under contaminated paddy conditions, particularly when Hg is present in more bioaccumulative forms. Therefore, the high BAFgrain observed in the unamended treatment previously exposed to 44 °C should be interpreted as evidence of substantial apparent Hg accumulation in edible tissue, but not as a direct indication of physiological translocation or Hg speciation.

These results indicate that total soil Hg alone was not sufficient to explain Hg accumulation in rice grains. Rather, grain Hg accumulation was likely influenced by the interaction among the operationally labile Hg fraction, substrate acidity, low organic matter content, sandy loam texture, flooded redox conditions, rhizosphere processes, dissolved organic ligands, and compost-mediated immobilization mechanisms [91]. Because this study quantified total Hg rather than methylmercury or inorganic Hg separately, the BAFgrain values represent apparent accumulation based on total Hg and should be interpreted cautiously. Future studies should include Hg speciation in grains and plant tissues to determine whether the high accumulation ratios are mainly associated with methylmercury formation, inorganic Hg uptake, or both.

The process of Hg accumulation in rice grain is complex and controlled by the interaction among substrate geochemistry, Hg speciation, flooding-induced redox conditions, rhizosphere processes, dissolved organic ligands, and plant development [92,93,94]. In rice-growing systems, flooding can promote anaerobic conditions that alter the mobility and transformation of Hg, particularly due to the activity of microorganisms associated with methylation processes. Although this study quantified total Hg rather than methylmercury, the literature indicates that contaminated rice paddies may constitute a significant route of human exposure to Hg species, due to the accumulation of the metal in the grain [92,95]. Furthermore, the Hg concentrations obtained in the grains exceed the maximum limit of 20 μg kg^−1^ established by the National Standard of the People’s Republic of China (2013), which is relevant from a public health perspective, given that rice is a frequently consumed food and chronic exposure to Hg through the diet may pose a risk to consumer populations, especially in regions where this cereal constitutes a staple food source.

In the present study, treatments without compost showed greater accumulation of Hg in the grain, particularly under conditions of prior thermal exposure at 40 and 44 °C. This response should be distinguished from the increase in the operationally labile Hg fraction in the substrate, because grain Hg accumulation did not follow a simple linear relationship with the maximum operationally labile Hg fraction. The highest labile Hg values occurred after exposure to 50 °C, but this condition did not produce harvestable grain and therefore could not be linked directly to Hg accumulation in edible tissue. Conversely, the highest grain Hg concentration occurred in S previously exposed to 44 °C. This mismatch indicates that Hg accumulation in grain depends not only on the labile Hg pool but also on plant establishment, reproductive development, substrate fertility, flooded redox dynamics, and the absence or presence of compost. Thus, the operationally labile Hg fraction should be interpreted as an indicator of potential Hg mobility in the substrate, whereas grain Hg concentration represents a later biological endpoint controlled by both Hg availability and crop performance.

Compost-amended treatments showed lower Hg concentrations in grains and lower BAFgrain values, suggesting a buffering effect of the amendment on the residual availability of the metal. However, this mitigating effect was not constant across the solarization conditions, as indicated by the compost × thermal exposure interaction. This effect can be attributed to processes of adsorption, complexation, ion exchange, chelation, and possible precipitation of Hg into less soluble forms—mechanisms widely described for organic amendments applied to metal-contaminated soils [19]. The presence of functional groups such as carboxyls, hydroxyls, and other reactive sites in organic matter is considered an important factor influencing Hg partitioning through the formation of Hg–ligand complexes, which may reduce Hg mobility and consequently limit Hg transfer to plants. Studies using organic amendments have consistently reported reductions in labile or operationally extractable Hg fractions and Hg accumulation in rice, although the magnitude of these effects depends on the type of amendment, its application rate, chemical composition, and the redox conditions of the system [96]. These processes are consistent with the lower increase in the labile Hg fraction and the lower BAFgrain values observed in compost-amended soils in the present study.

In addition to the direct immobilization of Hg, compost may have indirectly contributed to reducing its concentration in grain by improving substrate conditions and increasing plant growth. This effect can lead to dilution of the metal in edible tissue when biomass or grain production increases, a mechanism that has been reported in studies on the mitigation of Hg and MeHg in rice through organic amendments [97,98]. In the present study, this interpretation is consistent with the higher yield response observed in the SC treatments at 40 and 44 °C and with the lower Hg concentrations and BAFgrain values recorded in compost-amended treatments.

However, it is important to note that the response of organic amendments to Hg is not always unidirectional. Dissolved organic matter can form stable complexes that immobilize Hg, but it can also increase its mobility under certain conditions, depending on its aromaticity, molecular weight, degree of humification, and functional composition [19]. Likewise, in flooded systems, inputs of sulfur or labile organic matter can have dual effects: on the one hand, they can promote the precipitation of Hg as poorly soluble sulfides; on the other, they can stimulate reducing microbial communities capable of modifying Hg speciation [99]. Therefore, the reduction in grain Hg and BAFgrain observed in the compost treatments should be interpreted as the integrated result of immobilization processes, changes in the residual availability of the metal, and the crop’s yield response.

Overall, the results suggest that prior solarization-induced thermal exposure modified Hg dynamics in the substrate by increasing the operationally labile Hg fraction, but its translation into grain Hg accumulation depended on whether plant development and substrate conditions allowed Hg transfer to edible tissue. Compost application reduced grain Hg concentrations and BAFgrain values, supporting its potential as a mitigation strategy in mining-impacted soils. However, the compost × thermal exposure interaction demonstrates that this mitigating capacity may vary depending on the intensity of prior cover-induced solarization conditions. Given that the concentrations obtained exceeded international reference values for Hg in rice, the results highlight the need to assess the potential health risk associated with the consumption of rice grown on soils affected by gold mining.

### 3.8. Health Risk Due to Hg Exposure

The estimated weekly intake (*EWI*) and hazard quotient (*HQ*) values are presented in Table 4. *EWI* values did not exceed the historical provisional tolerable weekly intake (*PTWI*) for Hg of 4 μg kg^−1^ body weight week^−1^ established by JECFA [52], considering a weekly rice consumption rate of 1.374 kg week^−1^ for the department of Córdoba (ENAM) [34] and a reference body weight of 70 kg. The *HQ* values were below 1 in all treatments with available grain, suggesting no appreciable non-carcinogenic risk under the exposure assumptions considered. However, the highest *HQ* value was recorded in the unamended soil treatment (S) previously exposed to 44 °C, with a value close to 1, whereas the lowest values were observed in the compost-amended treatments (SC). These results are consistent with the lower Hg accumulation observed in rice grains from compost-amended substrates, suggesting that the amendment contributed to reducing the potential risk associated with grain consumption. Conversely, prior thermal exposure of the soil–amendment system tended to increase *HQ* values, mainly in the unamended treatments, which is consistent with the increase in the potentially bioavailable Hg fraction and its greater accumulation in rice grains under those conditions. Because this assessment was based on total Hg and did not quantify methylmercury, the results should be interpreted as a screening-level risk estimate rather than a definitive human health risk assessment.

Given the above, although *HQ* values were below 1, the Hg concentrations detected in rice grains, the high regional rice consumption, and the absence of methylmercury speciation justify maintaining monitoring, management, and mitigation strategies in rice-growing areas influenced by artisanal gold mining.

## 4. Conclusions

Prior solarization-induced thermal exposure of the soil–amendment system modified Hg dynamics in the evaluated substrates, increasing the operationally defined labile Hg fraction, interpreted here as an indicator of potential Hg mobility, especially after exposure to 50 °C. Although total Hg concentrations tended to decrease after the pretreatment, the increase in the labile fraction suggests a redistribution of Hg toward more extractable forms. However, because the thermal gradient was generated using plastic covers, the observed responses should be interpreted as the residual effects of cover-induced solarization conditions rather than as the effect of temperature alone.

The incorporation of compost reduced total Hg concentrations and partially attenuated the increase in the operationally labile Hg fraction, probably through adsorption, complexation, chelation, ion exchange, and immobilization processes associated with organic matter. Nevertheless, the mitigating effect of compost varied according to the intensity of prior solarization-induced thermal exposure and was less evident under the highest thermal condition.

Compost favored rice yield response under prior thermal exposures of 40 and 44 °C, but not under the reference condition of 32 °C. The absence of harvestable grains after exposure to 50 °C suggests that the most intense prior solarization condition may have generated residual negative effects on the capacity of the substrate to support the reproductive phase of the crop. Therefore, although the highest operationally labile Hg fraction was observed after exposure to 50 °C, this condition could not be directly linked to Hg accumulation in edible grain. Compost-amended treatments showed lower Hg concentrations in grains and lower soil-to-grain Hg bioaccumulation factors than unamended treatments, supporting the potential role of compost in reducing Hg accumulation in edible tissue. However, grain Hg concentrations exceeded the international reference limit for Hg in rice, indicating that rice produced on mining-impacted soils requires attention from a food safety perspective.

The highest grain Hg concentration and the highest BAFgrain value were observed in the unamended treatment previously exposed to 44 °C, indicating that Hg accumulation in rice grain was not explained by total soil Hg alone. Rather, grain Hg accumulation was likely influenced by the interaction among the operationally labile Hg fraction, substrate acidity, low organic matter content, sandy loam texture, flooded redox conditions, plant development, and the absence or presence of compost. The operationally labile Hg fraction should therefore be interpreted as an indicator of potential Hg mobility in the substrate, whereas grain Hg concentration represents a later biological endpoint controlled by both Hg availability and crop performance. Therefore, the BAFgrain values reported in this study should be interpreted as apparent soil-to-grain accumulation ratios and not as physiological translocation factors.

The *HQ* values were below 1 in all evaluated treatments with harvestable grain, although the unamended treatment previously exposed to 44 °C showed the highest value, close to 1. These results suggest no appreciable non-carcinogenic risk under the exposure assumptions considered. However, because only total Hg was quantified in grains and methylmercury was not determined, the risk assessment should be interpreted as a screening-level evaluation rather than as a definitive assessment of human health risk.

Overall, the results indicate that compost may be a useful amendment for attenuating Hg lability in pretreated substrates and reducing Hg accumulation in rice grains grown on mining-impacted soils. However, its effectiveness depends on the intensity of prior cover-induced solarization conditions and on the geochemical characteristics of the substrate. Future studies should evaluate Hg speciation, particularly methylmercury, as well as Hg in irrigation water, seeds, leachates, and interstitial water, microbial dynamics, Hg mass balance, and Hg distribution in roots, shoots, leaves, and grains under field conditions.

## Figures and Tables

**Table 1:** Characterization of compost made from contaminated plant biomass.

Variable	Units	NTC 5167	Compost
Characterization and composition of solid organic materials			
Moisture	%	Max 25	24.8
pH in saturation paste	pH range	Min 4–Max 9	7.25
Electrical Conductivity	dS m^−1^		3.1
Moisture Retention	%	Min 100	132.6
Ash	%	Max 60	16.6
Loss on Ignition	%		14.6
Cation Exchange Capacity	meq 100 g^−1^	Min 30	41
Apparent Density (dry basis)	g cm^−3^	Max 0.6	0.54
Total Oxidizable Organic Carbon	%	Min 15	15.9
Carbon/Nitrogen Ratio	N/A	Max 25	17.7
Characterization of the mineral fraction			
Total Nitrogen	%	Min 1	0.79
Organic Nitrogen	%		0.79
Total Phosphorus	%	Min 1	0.58
Total Potassium	%	Min 1	1.55
Total calcium	%		2.01
Total magnesium	%		1.19
Total sulfur	%		0.12
Total iron	%		1.78
Total manganese	mg kg^−1^		986
Total copper	mg kg^−1^		33.9
Total zinc	mg kg^−1^		106
Total boron	mg kg^−1^		175
Total sodium	%		0.09
Total silicon	%	Max 50	36.80
Acid-Insoluble Residue	%		42.10
Mercury	mg kg^−1^	Max 17	0.17

**Table 2:** Physicochemical parameters of the soils.

Code	Texture	pH	OM	S	P	Ca	Mg	K	Na	Al + H	ECEC
		1:1	%	mg kg^−1^		cmol^(+)^ kg^−1^					
S	Sandy loam	5.0 ± 0.3 ^a^	0.10 ± 0.02 ^a^	16.2 ± 0.5 ^a^	2.0 ± 0.2 ^a^	0.37 ± 0.04 ^a^	0.19 ± 0.03 ^a^	0.08 ± 0.01 ^a^	<0.11	2.42 ± 0.24	3.1 ± 0.3 ^a^
SC		7.3 ± 0.4 ^b^	1.87 ± 0.03 ^b^	50.5 ± 0.8 ^b^	24.1 ± 0.5 ^b^	7.94 ± 0.18 ^b^	2.95 ± 0.15 ^b^	1.62 ± 0.03 ^b^	0.40 ± 0.08	NA	18.2 ± 0.6 ^b^

S: soil without compost (control); SC: soil + compost; OM: organic matter. Note: Different letters indicate significant differences among treatments.

**Table 3:** Total and potentially bioavailable Hg concentrations in the experimental substrates.

	S	SC
	µg kg^−1^	
**Total Hg**	182.63 ± 38.82 **a**	125.64 ± 21.30 **b**
**Bioavailable Hg**	1.00 ± 0.04 **b**	1.25 ± 0.04 **a**

Different letters indicate statistically significant differences (*p* < 0.05) based on Welch’s independent-samples *t*-test. S: soil without compost (control); SC: soil + compost.

**Table 4:** Estimated weekly intake (*EWI*) and hazard quotient (*HQ*) of rice grown in mining-impacted soils after cover-induced prior thermal exposure, with and without compost amendment.

Code	*EWI* (μg kg^−1^ Body Weight Week^−1^)		*HQ*	
	S	SC	S	SC
32 °C	2.36 ± 0.13	1.22 ± 0.22	0.59 ± 0.03	0.31 ± 0.06
40 °C	2.50 ± 0.08	0.79 ± 0.16	0.62 ± 0.02	0.20 ± 0.04
44 °C	3.52 ± 0.55	0.93 ± 0.05	0.88 ± 0.14	0.23 ± 0.01

S: soil without compost (control); SC: soil + compost.

## Data Availability

The original contributions presented in this study are included in the article. Further inquiries can be directed to the corresponding author.
